# Knowledge and practice of nursing students regarding bioterrorism and emergency preparedness: comparison of the effects of simulations and workshop

**DOI:** 10.1186/s12912-022-00917-y

**Published:** 2022-06-14

**Authors:** Mahsa Ghahremani, Zahra Rooddehghan, Shokouh Varaei, Shima Haghani

**Affiliations:** School of Nursing and Midwifery, Tehran University of Medical Sciences, Tehran, Iran

**Keywords:** Training, Workshop method, Simulation, Bioterrorism, Nursing students

## Abstract

**Background and purpose:**

Bioterrorism is a global threat. Nurses are one of the first groups that should be ready for it. College years are the best time to educate these issues. This study aimed to compare the effectiveness of simulation and workshop on knowledge and practice of nursing students regarding bioterrorism.

**Materials and methods:**

This was an experimental study. The study sample consisted of 40 last-year nursing students who were randomly assigned to two groups by using random numbers table. Data was collected using a demographic questionnaire, bioterrorism knowledge scale, and an OSCE checklist. Before the intervention, the students completed the study questionnaires and a six-station OSCE test. The workshop group (20 students) learned how to deal with bioterrorism through lectures. The simulation group (20 students) participated in a simulation learning program. After one month, the students completed the study tools again. Finally, collected data were analyzed using descriptive and inferential statistics in SPSS V.16.

**Results:**

The difference between the knowledge and performance scores of both groups (workshop and simulation), before and after the intervention, was statistically significant (*P* < 0.001). Students in both groups had higher knowledge and performance scores after the intervention. The simulation group scores were higher than the workshop group scores in the knowledge and the most of performance domains.

**Conclusion:**

The simulation group had better results in terms of enhancing knowledge, preparedness, disaster triage, reporting, incident management, communication, mental disorders, and isolation domains compared to the workshop group.

**Supplementary Information:**

The online version contains supplementary material available at 10.1186/s12912-022-00917-y.

## Introduction

There is a considerable increase in the tendency to use biological agents since two decades ago. Bioterrorism aims to expand the damage to the public population [1]. Today, the dimensions of bioterrorism take a new shape by using the new technologies and the emergence of destructive nuclear weapons. It would cause unimaginable damages and high mortality in case of war [2]. Therefore, it is important to prepare disaster teams in this sector. They should be ready to reduce mortality from bioterrorism attacks which depend on access to efficient resources and forces [3].

Disaster management is introduced as one of the aspects of management in solving bioterrorism. Nurses, as one of the disaster team members, need to know the effects of bioterrorism on public health. Nurses can help in reducing anxiety and stress and prevent the spread of diseases if they receive the necessary education [4]. Despite the major role of nurses in responding to natural, human-made, and technological disasters, there is a shortage in this field in the content of nursing schools curricula. Also, educational programs in response to disasters have not been implemented properly or have failed to meet the standards of critical situations [5].

Education in this field must start from the treatment phase [4]. Nurses are at the forefront of treatment in the face of bioterrorism as they are the largest group of healthcare professionals [6, 7]. Meanwhile, nurses often do not have the required knowledge to deal with such phenomena [8]. Nurses are among carers who must be more aware of bioterrorism since it would increase the level of health security in the country. Various educational methods can be used to educate bioterrorism content to empower nurses in [5].

There are seven necessary skills for effective performance in face of disasters. The first required skill is the evaluation of the skill and knowledge of personnel about disasters [9]. The simulation method has strong validity and reliability for assessing clinical skills, compared to oral and written exams. This is because of its features such as its ability to create real-world situations without having their risks and its ability to assess educational objectives in cognitive, emotional, and psychomotor dimensions. While this method is very effective it is expensive [10, 11].

Educational simulation is an advanced technological method to promote knowledge and it is applied as a technique to achieve learning goals. This method is primarily used in the education field and showed a new clinical model in nursing education. During an educational simulation, instructors provide specific practice strategies for students to gain experience, solve problems and reduce the anxiety of real situations which can be caused by insufficient skills. Real situations can cause mental and physical damages and simulation can prevent them. The simulation provides a place for decision-making with no time and space restrictions [10, 12]. Due to the emergence of new infectious diseases such as AIDS, hepatitis, and tuberculosis, the knowledge of the medical staff and nurses need to be increased.

Given the essential role of nurses at the frontline of the healthcare system in dealing with bioterrorism and the importance of long-lasting education the present study aimed to compare the effectiveness of simulation and workshop on knowledge and practice of nursing students regarding bioterrorism.

## Materials and methods

This study was an experimental study that was conducted on last-year students in the seventh-semester BSc nursing students at the School of nursing and midwifery, Tehran University of Medical Sciences, Tehran, Iran. Forty students were selected by census sampling and randomly allocated by using random numbers table. Seven students were not included in the study because they did not want to participate. The inclusion criteria were being a BSc student, studying in the seventh semester, passing the emergency course, and providing written consent for participation in the study. Data were collected using a demographic characteristics questionnaire (age, gender, city of residence, nursing work experience, amount of time allocated to nursing work per week, marital status, history of facing a bioterrorism situation, and previous participation in educational courses), Emergency Preparedness Information Questionnaire (EPIQ), and six-station bioterrorism performance measurement checklists.

### Emergency preparedness information questionnaire (EPIQ)

The reliability of the questionnaire was estimated using internal consistency (α = 0.96). The questionnaire has 44 items, assessing nurses’ knowledge regarding activities needed during disaster response in eight areas including disaster triage, hospital incident command system (HICS), communication in disasters, isolation, disinfection and quarantine, mental disorders, epidemiology and decision-making, reporting, having access to vital sources, and recognition of biological factors. Each item is scored based on a five-point Likert (from not familiarized = 1 to completely familiarized = 5) [13].

### Bioterrorism performance measurement checklist

The bioterrorism performance measurement checklist is a researcher-made tool created based on the steps to bioterrorism. It is applied in the form of objective structured clinical examination (OSCE). It was approved by 10 faculty members and experts in the field of disaster management and passive defense. Each station included a scenario, a guide for the test, a guide for the test taker, an evaluation checklist, and a scenario for standard patients in case of using them. The validity of the checklists was confirmed by 10 faculty members and experts in the field of disaster management and passive defense. To assess the reliability of OSCE test checklists, two interviewers filled them for 10 cases simultaneously and Pearson’s correlation coefficient was r = 0.97.

#### The first week

All eligible students in both simulation and workshop groups who signed the informed consent form were asked to fill out the study tools. The knowledge of students was assessed using the questionnaire. A six-station OSCE test was used to assess the performance of study participants. Stations were designed based on six domains of bioterrorism preparedness. The performance was evaluated in each station with researcher-made checklists. In each station, one or some objectives were assessed. Standard patients, manikin, and required equipment were used based on the objectives in stations. The time for each station was 6 min with one minute for station changing. A coordination committee reviewed all stations and equipment one day before the test.

#### The second week

In 6, on a predetermined day, a faculty member who is an expert in the field of disaster management and passive defense presented all students in both simulation and workshop groups about bioterrorism, bioterrorism agents, and recognizing them.

#### The third week

In 7, students in the workshop group attended three hours workshop which was conducted using the lecture method. The contents were 1. Triage in disaster, 2. Hospital Incident Command System (HICS), 3. Communication in disasters, 4. Isolation, decontamination, and quarantine, 5. Mental health disorders, 6. Epidemiology and decision making, 7. Reporting and access to vital resources, and 8. Identification of biological factors. After the workshop, students had two hours to practice the content of the workshop. Students were not allowed to record or videotape the workshop to prevent contamination of the samples.

**Fig. 1:**
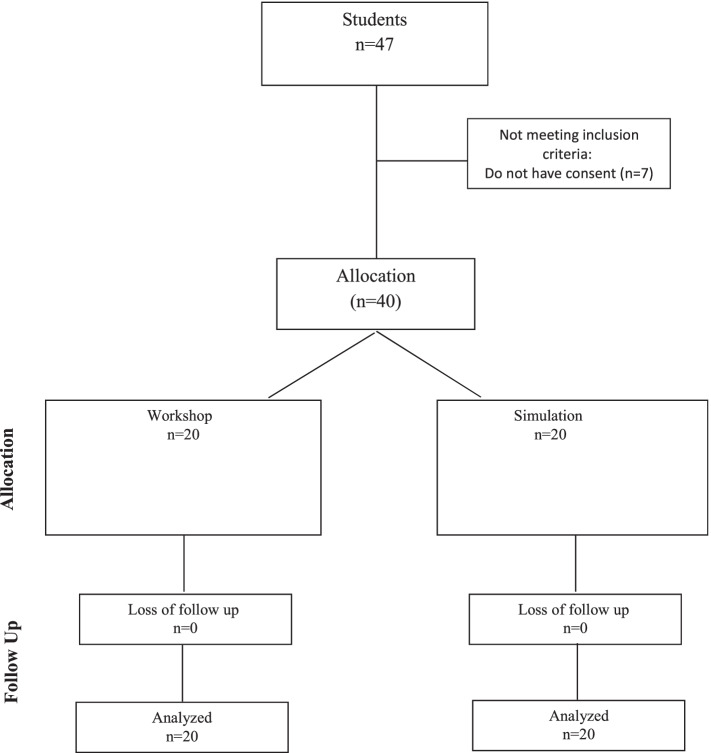
The process of sampling and allocation of study subjects

In the same week, the simulation group received a three-hour simulation based on a predetermined scenario. The content of the scenario was related to bioterrorism and all students played a role in it. All students practiced their roles under the supervision of the research team. Students were not allowed to record or videotape the simulation. At the end of the week, the students in the simulation team participated in a simulation of a bioterrorism incident. In the fourth week, all participants completed research tools and attended the six-station OSCE. The process of sampling and allocation of study subjects is presented in Fig. 1.

The study tools were completed before the education and after one month.

#### Data analysis

Data analysis was performed using SPSS version 16. Descriptive (absolute and relative frequency, mean and standard deviation) and inferential (independent t-test, paired t-test, Chi-square) statistics were used. A P-value less than 0.05 was considered statistically significant. Data is attached as a supplementary file.

#### Ethical considerations

The research was approved by the joint ethics committee and the vice-chancellor for research of the school of nursing, midwifery, and rehabilitation of Tehran University of Medical Sciences (IR.TUMS.FNM.1397.081). The participants were ensured of confidentiality regarding their personal information.

## Results

In this study, the majority of the subjects in the simulation group (65%) were female, while half of the participants in the workshop group were male (chi = 0.921, *p* = 0.337). The mean age of the simulation and workshop groups was 23.95 ± 3.70 and 23.353.09, respectively (t = 0.555, *p* = 0.582). None of the students in the study had been exposed to bioterrorism and had not completed courses related to bioterrorism. The Mann-Witney U test showed a significant difference in the knowledge scores of the workshop group before and one month after the intervention (*P* < 0.001).

There was a significant difference in the performance of study subjects in the communication, incident command, and reporting domains before and after the intervention. There was a significant increase in the performance scores after the intervention in these domains. However, no significant difference was observed in the triage, isolation, and mental disorders domains in the simulation group before and after the intervention. The mean score increased after the intervention but the increase was not significant (Table 1). The performance score of students in the simulation group was changed significantly. Also, there were significant differences between the two groups' scores.

**Table 1:** Nursing students' knowledge and performance score before and after intervention in simulation and workshop groups

Variables	Group	Before intervention	After intervention	Test results Wilcoxon
		Mean ± SD	Mean ± SD	
EPIQ	Simulation	88.15 ± 36.96	74.25 ± 35.29	Z = -3.884, *p* = 0.001
	Workshop	156.20 ± 12.73	124.95 ± 13.40	Z = -3.825, *p* = 0.001
	Mann-Witney U	Z = -1.083, *p* = 0.289	Z = -4.793, *p* = 0.001	
Disaster triage	Simulation	10.15 ± 1.84	12.25 ± 4.88	Z = -0.884, *p* = 0.377
	Workshop	8.45 ± 2.64	9.75 ± 3.79	Z = -1.402, *p* = 0.161
	Mann-Witney U	Z = -2.130, *p* = 0.035	Z = -1.747, *p* = 0.086	
Comunication	Simulation	0.55 ± 0.75	7.85 ± 1.08	Z = -4.025, *p* = 0.001
	Workshop	0.40 ± 0.68	5.65 ± 1.75	Z = -3.967, *p* = 0.001
	Mann-Witney U	Z = -0.673, *p* = 0.583	Z = -3.657, *p* = 0.001	
Command System	Simulation	3.80 ± 1.88	15.90 ± 4.38	Z = -3.941, *p* = 0.001
	Workshop	4.25 ± 4.92	11.75 ± 2.04	Z = -3.398, *p* = 0.001
	Mann-Witney U	Z = -0.508, *p* = 0.620	Z = -3.747, *p* = 0.001	
Isolation	Simulation	4.15 ± 0.74	7.60 ± 1.66	-3.801, *p* = 0.001
	Workshop	3.10 ± 1.44	6.45 ± 1.19	Z = -3.845, *p* = 0.001
	Mann-Witney U	Z = -2.302, *p* = 0.026	Z = -3.034, *p* = 0.002	
Psychopathy	Simulation	5.80 ± 2.68	8.95 ± 1.95	Z = -3.434, *p* = 0.001
	Workshop	5.55 ± 2.11	6.80 ± 1.85	Z = -2.378, *p* = 0.017
	Mann-Witney U	Z = -0.685, *p* = 0.512	Z = -3.294, *p* = 0.001	
Report	Simulation	7.50 ± 2.80	14.05 ± 3.95	Z = -3.831, *p* = 0.001
	Workshop	6.45 ± 4.48	7.05 ± 2.72	Z = -0.545, *p* = 0.586
	Mann-Witney U	Z = -1.430, *p* = 0.157	Z = -4.658, *p* = 0.001	

The comparison of study scales before and after intervention between males and females showed that female students had better scores in disaster triage than male students before the intervention. After the intervention, both genders showed similar results in the disaster triage domain. After the intervention females had higher scores in command System and isolation domains (Table 2).

**Table 2:** The comparison of nursing students' knowledge and performance Score before and after Intervention in simulation and workshop groups based on gender

Variables	Gender	Before intervention	After intervention
		Mean ± SD	Mean ± SD
EPIQ	Female	80.07 ± 30.39	154.61 ± 13.25
	Male	103.14 ± 45.55	159.14 ± 12.11
	Mann–Whitney U	Z = -0.055, p = 0.957	Z = -0.383, *p* = 0.705
Disaster triage	Female	11.07 ± 1.03	12.07 ± 4.27
	Male	8.42 ± 1.81	12.57 ± 6.24
	Mann–Whitney U	Z = -2.613, *p* = 0.009	Z = -1.339, *p* = 0.191
Communication	Female	0.53 ± 0.87	7.69 ± 0.85
	Male	0.57 ± 0.53	8.14 ± 1.46
	Mann–Whitney U	Z = -0.908 *p* = 0.448	Z = -0.280, *p* = 0.787
Command System	Female	3.46 ± 1.76	17.23 ± 4.62
	Male	4.42 ± 2.07	13.42 ± 2.69
	Mann–Whitney U	Z = -0.361, *p* = 0.725	Z = -2.191, *p* = 0.030
Isolation	Female	4.38 ± 0.76	8.07 ± 1.38
	Male	3.71 ± 0.48	6.71 ± 1.88
	Mann–Whitney U	Z = -2.002, *p* = 0.055	Z = -2.424, *p* = 0.017
Psychopathy	Female	5.30 ± 2.98	8.76 ± 2.38
	Male	6.71 ± 1.88	9.28 ± 0.75
	Mann–Whitney U	Z = -0.083, *p* = 0.935	Z = -0.494, *p* = 0.645
Report	Female	7.46 ± 3.25	15.38 ± 3.59
	Male	7.57 ± 1.90	11.57 ± 3.55
	Mann–Whitney U	Z = -0.537, *p* = 0.607	Z = -1.470, *p* = 0.149

## Discussion

In this study, nursing students’ knowledge and practice regarding bioterrorism was compared before and one month after the intervention in the workshop and simulation groups. There was a significant difference in the mean scores in the workshop group before and after the intervention. Our results showed that nursing students had low knowledge before the intervention in both groups. The previous literature also revealed that most nurses had limited knowledge about bioterrorism. The results of a study on 291 nurses, physicians, nursing students, and medical students showed that the knowledge scores of the respondents were low. Respondents answered correctly to less than one fourth of the knowledge questions. Also, less than 23% of the respondents stated confidence to provide health care in an assumed chemical terrorism situation. The findings showed a need for healthcare providers in continuing education to develop, implement, and evaluate new local terrorism preparedness programs [14].

The results of a cross-sectional study in Saudi Arabia on 1030 participants showed that mean knowledge score of bioterrorism-related agents was 4.92 ± 1.86 out of 12 points which was low. Respondents who had received previous training in bioterrorism preparedness had a significantly higher score in perceived benefits of education than those not sure and without prior training [15]. The results of a study in Poland showed that to assess level of nurse’s knowledge of bioterrorist hazard in Poland and need for additional training in this field. The Results showed that 87% of the nurses stated that their knowledge of biological warfare was not sufficient, Also, 92% of them understood the need to organize trainings for medical staff on bioterrorism threat and procedures in case of such an attack [16].

In a study by Bork and Rega [17], the mean percentage of nurses' knowledge of botulism in the disease control center was estimated at 74.25%. Another research showed that before education, most nurses in the operating room did not have enough ability to respond to bioterrorism incidents. After training, there was an increase in their level of preparedness in this regard [19].

According to the results of this study, the mean score of the participants’ knowledge of bioterrorism was low and moderate before the intervention. The study subjects had low knowledge regarding bioterrorism, and more than 65% of the responders reported poor knowledge. In a study that was conducted by Gorji, Niknam [18], 91.7% of nurses had low knowledge of bioterrorism, whereas 93.3% of the students had no attitude toward it. The educational workshop had a positive impact on the critical thinking of the subjects, and the mean critical thinking score of the nursing students was improved after the workshop [19]. A study by Aghaei and Nesami [8] was conducted to assess the effect of bioterrorism education on knowledge and attitudes of nurses. They selected 65 nurses and their results showed that before the educational intervention the knowledge about bioterrorism of majority of nurses was low. After education, the knowledge about bioterrorism of majority of subjects had been increased. The results of another study which was conducted to compare a computerized bioterrorism versus a standard bioterrorism education and training program on the participants’ responses to biological agents showed that both programs improved participants’ ability [20]. Atakro, Addo [21] assessed and compared the knowledge, attitudes, and preparedness of emergency department nurses and medical officers concerning potential bioterrorist attacks in Ghana. Their results showed that bioterrorism knowledge between emergency department nurses and emergency department MOs was different. Emergency department nurses and MOs were unprepared for these kind of attacks. They had positive attitudes toward bioterrorism preparedness education.

Given the lack of training programs, which is a major obstacle, it is recommended that more effective training methods can be provided using multidisciplinary simulation approaches. Disasters are unpredictable and preventing them is impossible, emergency preparation systems that provide disaster preparation and incident response activities can minimize the adverse outcomes of such incidents [22]. Concerning the importance of education in improving nurses' readiness to deal with natural disasters and incidents, implementing these courses in a variety of ways (e.g., workshops, conferences, and movies) seems to be necessary. Among university students of disciplines that did not have bioterrorism in their curriculum, there were no significant differences between students regarding bioterrorism knowledge and preparedness score. Given the medium and low level of bioterrorism knowledge and preparedness of students, it is necessary to enhance this issue by modifying healthcare-related disciplines curricula.

Our results showed that female students had better scores in disaster triage than male students before the intervention. After the intervention, both genders showed similar results in the disaster triage domain. After the intervention females had higher scores in command System and isolation domains. The results of the study by Hamzeh pour and Khajehnasiri [23] showed that while the mean score of knowledge of male and female students was similar before the intervention the scores increased more significantly in female students. The changes in the scores may be related to the higher engagement of female students in the courses and their higher scores. However in our study after the course their scores were similar.

## Limitations

One of the major limitations of the present study was the lack of cooperation of students with the researcher despite substantial privileges, coordination of time, conducting OSCE test and designing stations, as well as lack of facilities and equipment for training. The problem of equipment shortages was eliminated by consulting with schools and hospitals that have already conducted bioterrorism preparation workshops. To encourage students to participate in the study, four days of the internship of students were reduced in coordination with the vice-chancellor for education, the supervisor of the internal-surgery department, and the special supervisor. Moreover, there was no need to pass the passive defense workshop as a part of the curriculum, and participation in studies was considered a workshop. The necessary guidance was provided by the experts in the field of crisis management at the university to design the stations properly.

## Conclusion

Based on our results the simulation group had better results in terms of enhancing knowledge, preparedness, disaster triage, reporting, incident management, communication, mental disorders, and isolation domains compared to the workshop group. We recommend to maintain knowledge and skills in bioterrorism management using courses in the form of workshops and simulations for nurses. We also suggest using simulation to educate bioterrorism management in universities and continuous education. Also, this training should be repeated periodically for nurses.

## Supplementary Information

**Additional file 1.**

**Additional file 2.**

## Data Availability

All data generated or analysed during this study are included in this published article [and its supplementary information files].
