# Supplementary figures and images for: Knowledge and practice of nursing students regarding bioterrorism and emergency preparedness: comparison of the effects of simulations and workshop
Source: BMC Nurs. 2022 Jun 14;21:152. doi: 10.1186/s12912-022-00917-y (PMC9195329; doi:10.1186/s12912-022-00917-y)

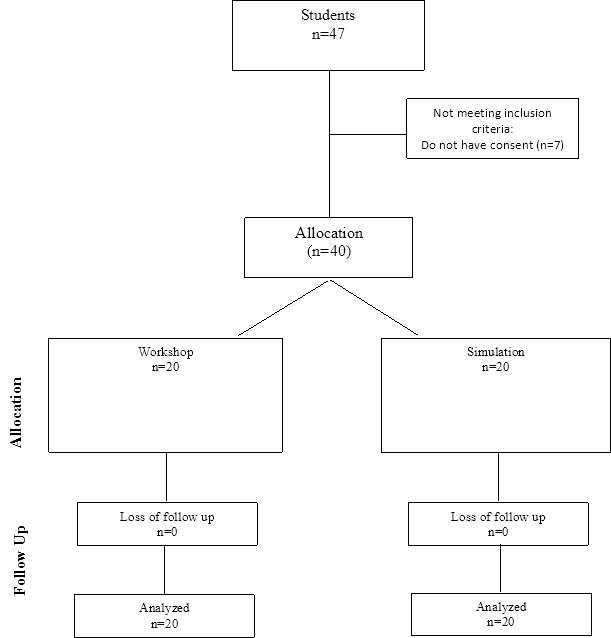

Supplement: Supplementary file 2 — Additional file 2. [file 12912_2022_917_MOESM2_ESM.jpeg]
